# Comprehensive Assessment of Cannabidiol and HU308 in Acute and Chronic Colitis Models: Efficacy, Safety, and Mechanistic Innovations

**DOI:** 10.3390/cells13232013

**Published:** 2024-12-05

**Authors:** Dinesh Thapa, Mohan Patil, Leon N Warne, Rodrigo Carlessi, Marco Falasca

**Affiliations:** 1 Curtin Health Innovation Research Institute (CHIRI), Curtin University, Perth, WA 6102, Australia; mohan.patil@postgrad.curtin.edu.au (M.P.); leon.warne@anaesthesia.vet (L.N.W.); rodrigo.carlessi@curtin.edu.au (R.C.); 2 College of Science, Health, Engineering and Education, Murdoch University, Perth, WA 6150, Australia; 3 Harry Perkins Institute of Medical Research, QEII Medical Centre and Centre for Medical Research, The University of Western Australia, Nedlands, WA 6009, Australia; 4 Department of Medicine and Surgery, University of Parma, 43125 Parma, Italy

**Keywords:** inflammatory bowel disease (IBD), ulcerative colitis, inflammation, cannabidiol (CBD), cannabinoid 2 receptor, HU308

## Abstract

Cannabinoids are emerging as promising treatments for inflammatory diseases such as ulcerative colitis. Specifically, cannabinoid 2 (CB2) receptors, which are upregulated during inflammation, have been distinctively linked to anti-inflammatory and analgesic effects. HU308, a synthetic cannabinoid developed to activate CB2 receptors selectively, aims to minimize unwanted off-target side effects. This study evaluated the effectiveness of both cannabidiol (CBD) and HU308 in mouse models of dextran sodium sulphate (DSS)-induced colitis, which mimic the acute and chronic phases of ulcerative colitis. Mice were treated with DSS in drinking water (four percent for the acute model and one to two percent for the chronic model) to induce colitis, as indicated by increased disease activity index (DAI) scores and inflammatory markers. Treatment with 60 mg/kg of CBD, but not lower doses, significantly reduced colitis symptoms, such as inflammation, cytokine levels, and MPO activity, while also normalizing glucagon-like peptide-1 (GLP-1) levels. HU308 showed comparable efficacy to high-dose CBD (60 mg/kg) but at a much lower dose (2.5 mg/kg), without observable toxicity. HU308 effectively normalized DAI scores, colon inflammation, ammonia levels, and GLP-1 expression in both colitis models. These results suggest that both CBD and HU308 are promising treatments for ulcerative colitis. However, HU308 demonstrates enhanced therapeutic potential by achieving similar outcomes at a fraction of the dose required for CBD, reducing the risk of off-target side effects. The ability of HU308 to modulate GLP-1, a biomarker of gut endocrine function, further underscores its promise as a novel treatment option.

## 1. Introduction

Ulcerative colitis (UC) is a chronic, relapsing disorder of the gastrointestinal (GI) tract marked by excessive inflammation of the colon and tissue damage [1]. Emerging evidence indicates that UC extends beyond the GI tract, presenting with extraintestinal manifestations that complicate specific treatment development [2,3]. Conventional pharmacological therapies provide some relief but are often accompanied by significant side effects and inconsistent efficacy [4]. New therapies, including biologics such as anti-tumour necrosis factor-alpha (anti-TNF-α) and monoclonal antibodies (e.g., anti-integrins and anti-interleukins such as IL-12/23), show better clinical efficacy but remain inaccessible to the general population due to high costs, necessitating the exploration of novel therapeutic strategies [5,6].

Cannabinoids are reported to have analgesic and anti-inflammatory effects in several preclinical models including gastrointestinal and ocular disorders [7,8,9]. Cannabidiol (CBD), a non-psychoactive phytocannabinoid derived from *Cannabis sativa*, has garnered considerable attention for its potential as a therapeutic agent in pain and inflammatory conditions, including UC [7,10,11]. Despite promising preclinical results, clinical trials have not shown CBD has significant efficacy in UC treatment [9]. This discrepancy may be attributed to CBD’s complex pharmacology, as it interacts with multiple receptors within the endocannabinoidome, a lipid signalling system comprising canonical and non-canonical cannabinoid receptors, endogenous ligands, and their metabolizing enzymes [12,13].

The cannabinoid 2 (CB2) receptor, one of two canonical cannabinoid receptors, is significantly upregulated in the GI tract, particularly in the colon, during UC [14,15]. CBD’s pharmacological effects are reported to be mediated by the modulation of multiple receptors, including CB2, G protein-coupled receptor 55 (GPR55), and 5-HT1A receptors [12]. However, this multi-target interaction may lead to off-target effects, potentially limiting CBD’s clinical utility [12,16].

Given the anti-inflammatory and analgesic effects associated with CB2 receptor activation [7], there is a strong rationale for developing selective CB2 agonists as targeted therapy for UC. HU308, a synthetic CBD analogue, was designed to selectively target the CB2 receptor [17,18]. HU308 has demonstrated analgesic and anti-inflammatory effects in several preclinical models of pain and inflammation by selectively activating CB2 receptors [7,19,20,21]. The selective activation of CB2 receptors by HU308 offers the potential for enhanced efficacy while minimizing the risk of off-target effects, thus addressing a critical need in UC therapeutics.

This study aims to assess the pharmacological efficacy of CBD and HU308 in treating UC using acute and chronic mouse models of dextran sodium sulphate (DSS)-induced colitis, which mimics human colitis. Through a comprehensive evaluation of disease activity indices, histopathological changes, and inflammatory markers, we seek to elucidate the therapeutic potential of HU308 compared to CBD in UC management. Our findings will provide valuable insights into the comparative efficacy and safety profiles of these compounds, potentially advancing the development of CB2 receptor-targeted therapies, such as HU308, for UC treatment.

## 2. Materials and Methods

### 2.1. Animal Care and Use

All animal care and experiments complied with the Australian Code and NZ Guide for welfare issues relating to animal use in research and teaching. They were approved by the Curtin University Animal Research Ethics Committee (approval number: ARE2022-20). Animal use was in accordance with the Australian Code for the Care and Use of Animals for Scientific Purposes 8th Edition 2013 (updated 2021). Female BALB/c mice aged 8–12 weeks (ARC and Ozgene, Perth, Australia) were used for the experiments following a one-week period of acclimatization in specific pathogen-free (SPF) conditions with controlled temperature, humidity, and a 12 h light/dark cycle. Mice were housed at a maximum of 5 per cage and were provided standard laboratory chow diet and water ad libitum.

### 2.2. Dextran Sodium Sulphate Solution Preparation

Dextran sulphate sodium of colitis grade (MW 36,000–50,000 Da, Lot-No. S8634; MP Biomedicals, Santa Ana, CA, USA) was used to make a 4% (*w*/*v*) concentration solution in autoclaved drinking water to administer to the mice.

### 2.3. Induction of Colitis

A previously reported refined and translationally relevant model of acute and chronic colitis was used [22], which is briefly discussed below.

#### 2.3.1. Acute Colitis

Acute colitis was induced by challenging the mice with 4% DSS solution as the sole source of drinking water for seven consecutive days followed by regular normal drinking water (autoclaved) for three days. Freshly prepared 4% DSS solution is changed every three days. Control mice received normal autoclaved drinking water throughout the experiment.

#### 2.3.2. Chronic Colitis

Chronic colitis was induced over 24 days with 2% DSS water for the first seven days, followed by 1% DSS for 10 days (days 8–17) and 2% DSS for another seven days (days 18–24). Freshly prepared DSS solution is changed every three days. The change in concentration of DSS is to reflect the relapse and remission phase of the disease, like in human colitis. Control mice received normal drinking water throughout the experiment.

### 2.4. Experimental Design and Pharmacological Treatments

#### 2.4.1. Experimental Design

The experiments were designed by randomly allocating mice into healthy control groups, DSS vehicle, and treatment groups. Treatments and data collection were blinded to minimize bias and ensure the validity of the results.

#### 2.4.2. Pharmacological Treatments for Acute Colitis

**Figure 1:**
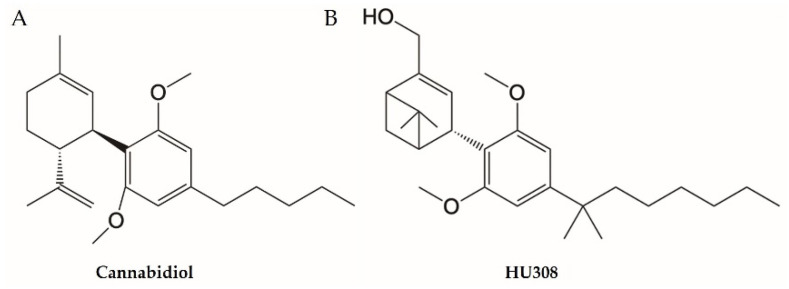
Chemical structure of cannabidiol (**A**) and HU308 (**B**).

Mice were randomly divided into healthy control, vehicle, and drug treatment groups (*n* = 5–8/group). CBD (Commonwealth Extracts, Louisville, KY, USA; Figure 1A) and HU308 (Axon Medchem BV, 9700 AT, Groningen, The Netherlands; Figure 1B) were administered intraperitoneally (i.p.) at a volume of 10 mL/kg once daily in 1:1:18 mixture of dimethyl sulfoxide (DMSO; Sigma-Aldrich, St. Louis, MO, USA), Tween80 ^®^ (Sigma-Aldrich, St. Louis, MO, USA), and normal saline, respectively. CBD was administered at doses of 10, 30, or 60 mg/kg, while HU308 was administered at doses of 1, 2.5, or 10 mg/kg. Treatment was initiated concurrently with DSS exposure, i.e., from day 1 and continued till the end of the study (day 11). Mice were sacrificed on day 11.

#### 2.4.3. Pharmacological Treatments for Chronic Colitis

Mice were randomly divided into healthy control, vehicle, and drug treatment groups (*n* = 8/group). Treatments were given in two phases. The first phase includes the period when mice received a 2% DSS challenge, i.e., from day 1 to day 7, and the second phase includes the period from day 18 to day 24. Treatments were paused when they were in 1% DSS (days 8–17). Colitis mice were treated either with vehicle or 2.5 mg/kg HU308 i.p. at a volume of 10 mL/kg. Mice were sacrificed on day 24.

### 2.5. Assessment of Colitis Clinical Markers

#### 2.5.1. Disease Activity Index Score

The Disease Activity Index (DAI) score provides an evaluation of the severity of colonic inflammation, considering three primary clinical parameters: body weight loss, stool consistency, and the presence of faecal occult blood or rectal bleeding. Additionally, other clinical indicators of colitis, such as alterations in behaviour and signs of pain and distress (e.g., hunched posture, reduced grooming, and decreased locomotor activity) were also recorded. The DAI score was determined using the previously established protocol [22], as summarized in the table below (Table 1).

#### 2.5.2. Pain Behaviours

Small lab animals show signs of pain usually through facial expressions. It is usually challenging to standardize such pain behaviours. Therefore, the Grimace Scoring Method (GSM), a well-established non-invasive and objective way to evaluate pain behaviours in laboratory mice, is used in this study to assess pain behaviours [23]. Mice were observed daily for 5 specific facial features: orbital tightening, nose bulge, whisker change, cheek bulge, and ear position. Additionally, changes in body position and movement were assessed as overall indicators of pain or discomfort. Table 2 outlines the criteria used to score pain-related behaviours in the mice.

### 2.6. Random Blood Glucose

On the final day of the experiments, glucose was measured in tail-pricked blood using a handheld blood glucometer (Accu-chek Performa^®^, Roche Ltd., Basel, Switzerland). Random blood glucose was measured in all groups including normal control, vehicle, and drug-treated colitis groups to see if colitis and the treatments impacted blood glucose levels. Blood glucose levels are expressed in mmol/L.

### 2.7. Euthanasia and Tissue Collection

At the end of the experiments, on day 11 for acute and day 24 for chronic disease, mice were anaesthetized using 2.5–3% isoflurane in the presence of oxygen (1 L/min) and the blood was collected using cardiac puncture. Following cardiac puncture, mice were euthanized (cervical dislocation), and colon, spleen, liver, and kidney tissues were collected, cleaned with cold 1% phosphate buffer saline (PBS; Sigma-Aldrich, St. Louis, MO, USA), and assessed for different markers of DSS-induced colitis changes.

#### 2.7.1. Colon Length

One of the prominent macroscopic features of DSS-induced colitis is the shortening of colon length. Reduced colon length indicates the severity of colonic inflammation and damage. In colitis, the colon length is shown to decrease significantly [22]. Therefore, we measured the length of the colon in all experimental groups to see if our treatments increased the colon length in comparison to the DSS-induced colitis group. The following protocol was used to measure colon length. Following euthanasia, the entire colon, from the cecum to the rectum, is carefully removed. The colon is then placed on a clean, flat surface with paper towel. The length from the caecal tip (the beginning of proximal colon) to the rectum (the end of the distal colon) was measured using a ruler, and images were captured. To ensure an accurate measurement of the colon’s true length, imaging was performed immediately after isolation from the animals. The colon lengths of all the experimental groups were measured using the same conditions, recorded, and analysed. Following the measurement, the colon is thoroughly washed with cold 1% PBS using an oral gavage needle-fitted syringe. A small portion (~1 cm) of the mid colon was kept in formalin for histological assessments and the rest of the remaining colon was snap-frozen in liquid nitrogen and stored at −80 °C for further analysis.

#### 2.7.2. Organ to Body Weight Percentage

It has been reported that, apart from colon inflammation, colitis presents extraintestinal manifestations. Therefore, to see the impact of colitis on other organs, vital organs including the liver, spleen, and kidney were carefully removed from animals, paper-dried, and weighed. The percentage of the relative weight of these organs based on their body weight was compared and analysed among different groups.

#### 2.7.3. Myeloperoxidase Activity

Myeloperoxidase, an enzyme predominantly found in specific white blood cells, particularly neutrophilic granulocytes, plays a pivotal role in the immune system. Neutrophils, recognized as the hallmark of acute inflammation, are essential in combating foreign pathogens. Therefore, evaluating MPO levels during colitis provides valuable insights into the intensity of the immune response and the efficacy of drugs targeting inflammation and immune response. MPO activity directly corresponds to the number of active neutrophil granulocytes, as MPO is stored in the granules within neutrophils and released upon degranulation. MPO activity in colon and spleen tissues was measured using a previously reported protocol [24,25,26]. Briefly, colon and spleen samples were collected in a standardized manner, blotted dry, weighed, and placed in a potassium phosphate buffer (pH = 6.0) containing 0.5% hexadecyltrimethylammonium bromide (HTAB; Sigma-Aldrich, St. Louis, MO, USA) at a ratio of 100 mL per 5 g tissue. The samples were manually crushed, homogenized, and sonicated. Subsequently, the homogenate was centrifuged for 15 min at maximum speed and 4 °C, and the supernatant was collected. A volume of 7 μL of the supernatant was added to 200 μL of 50 mM potassium phosphate buffer (pH 6.0) containing 0.167 mg/mL of O-dianisidine hydrochloride and 0.5 μL of 1% H_2_O_2_/mL. The kinetics of MPO activity were measured at 460 nm by recording changes in absorbance over 1 min using an automated multimode 96-well plate reader (PerkinElmer, Hong Kong, China).

### 2.8. Determination of Pro-Inflammatory and Anti-Inflammatory Cytokines in Colon Tissues

To measure the extent of colon inflammation, concentrations of different pro-inflammatory cytokines, including TNFα, IL-1β, IL-6, IL-12, and CCL2 (MCP-1), and anti-inflammatory cytokine (IL-10) were quantified in colon tissue samples using a mouse cytokine/chemokine multiplex assay kit (MCYTOMAG-70K, EMD Millipore, Darmstadt, Germany). Briefly, −80 °C frozen colon tissues were homogenized in radioimmunoprecipitation (RIPA) buffer (Sigma-Aldrich, St. Louis, MO, USA), sonicated, and centrifuged for 15 min at maximum speed. The supernatant was collected, and protein concentration was estimated using a commercially available Micro BCA assay kit (ThermoFisher Scientific, Waltham, MA, USA) to ensure equal loading of the protein sample. The assay was performed using the manual instructions provided by the manufacturer. Readings were obtained on an automated MagPix Luminex100 reader (Luminex Corporation 12212 Technology Blvd. Austin, TX, USA), and analysed using Software xPONENT^®^ 4.3 version. Cytokine concentrations were expressed as pg/mg of protein.

### 2.9. Colon Histopathology

Changes in colon histopathology were assessed using hematoxylin and eosin (H and E) staining as described previously [27]. Briefly, mouse colon samples were fixed in 10% normal buffered formalin (NBF; Sigma-Aldrich, St. Louis, MO, USA) and processed to paraffin wax on a 3.5 h cycle (dehydrated, cleared, and infiltrated in wax) using the Leica Peloris Rapid Tissue Processor (Leica Biosystems, Deer Park, IL, USA). The tissue was embedded in Paraplast paraffin wax, and full-faced sections at 4 µm were obtained using a Leica Histocore Biocut Microtome. Slides were dried overnight and stained with Hematoxylin and Eosin using the Leica ST5010 XL Auto stainer (Leica Biosystems, Deer Park, IL, USA). Slides were then covered and slipped in CV Mount using the Leica CV5030 cover slipper (Leica Biosystems, Deer Park, IL, USA) and scanned to obtain images (40×) under an automated AxioScan Z.1 microscope (Carl Zeiss GmbH, Jena, Germany). Treatment-blinded coded images were analysed using the scoring parameters (Table 3) reported previously [28,29,30].

### 2.10. Plasma and Colon GLP-1 Measurement

DSS-induced colon damage was measured by assessing the total glucagon-like peptide-1 (GLP-1) levels in colon lysates and plasma samples using a commercially available GLP-1 ELISA kit (EMD Millipore, Darmstadt, Germany) as a marker to check the effectiveness of our treatments. GLP-1 is a multifaceted biomarker of gut endocrine function, reflecting key aspects of gut health, inflammation, and epithelial repair. It serves as an indirect indicator of both localized and systemic therapeutic effects. Beyond its well-established roles in glucose metabolism and weight regulation, GLP-1 has been shown to reduce pro-inflammatory cytokine levels and enhance gut barrier integrity, both of which are critical in the context of colitis [31]. As GLP-1 levels are often dysregulated in colitis, we sought to evaluate whether our treatments could normalize GLP-1 levels to those observed in healthy animals, thereby providing insight into the potential efficacy of the intervention. The manufacturer-provided manual instructions were followed to carry out the assay, and readings were obtained on an automated multimode 96-well plate reader (PerkinElmer, Hongkong, China). The values were expressed in picomolar (pmol) concentrations for plasma samples and pmol/µg of protein for colon lysates.

### 2.11. Complete Blood Count and Plasma Biochemistry

Fresh whole blood collected in heparin-coated tubes (Element DC, Heska, Loveland, CO, USA) was used to measure blood haematological parameters using an automated blood analyser machine (BC-2800, Mindray, Shenzhen, China). Biochemical parameters including alanine aminotransferase (ALT), aspartate aminotransferase (AST), blood urea nitrogen (BUN), albumin, globulin, creatinine, ammonia, and total protein were measured in plasma in an automated biochemistry analyser (Element DC, Heska, Loveland, CO, USA).

### 2.12. Statistical Analysis

Statistical analysis was performed using GraphPad Prism software version 8 (San Diego, CA, USA). Data are presented as mean ± standard error of the mean (SEM). The significance of differences between groups was determined using *t*-tests or one-way analysis of variance (ANOVA) followed by Dunnett’s multiple comparisons post hoc test as appropriate. The symbol “#” is used to compare the significance between healthy control groups and the vehicle-treated colitis, whereas “*” is used to compare the drug treatments with vehicle colitis. Values of *p* < 0.05 were considered statistically significant.

## 3. Results

### 3.1. Results of Acute Studies

#### 3.1.1. Impact of CBD and HU308 Treatments on DSS-Induced Clinical Scores

##### CBD and HU308 Reduced DAI Scores

**Figure 2:**
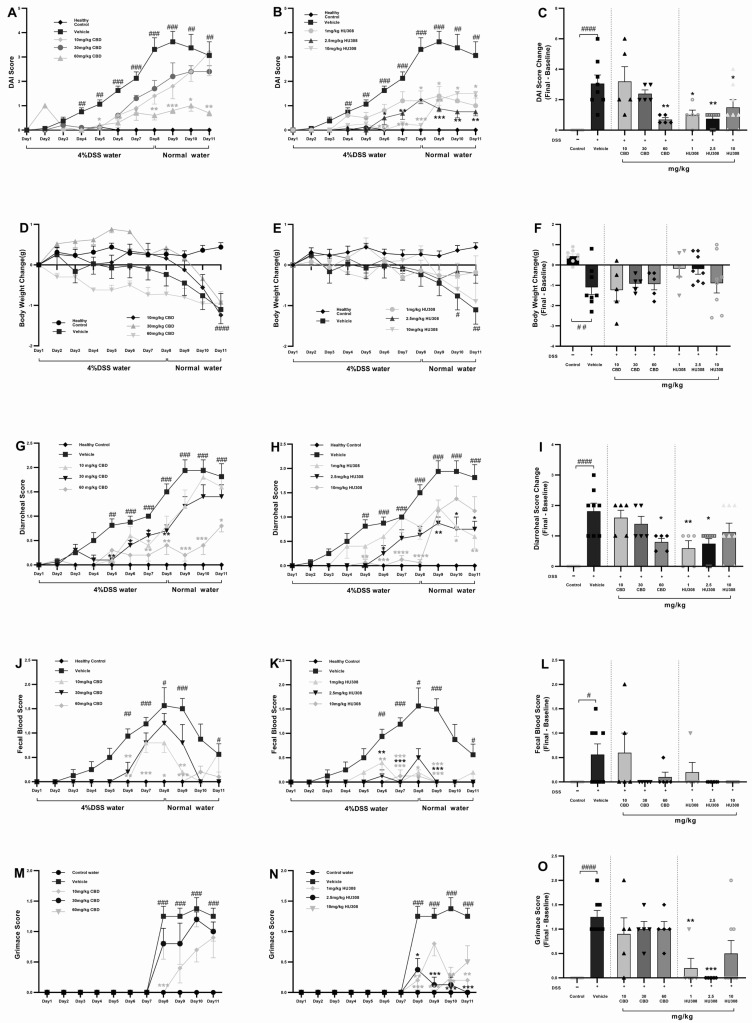
**Clinical scores in DSS-induced colitis with CBD and HU308 treatments**. (**A**,**B**) Daily Disease Activity Index (DAI) scores. (**C**) Final DAI score change on day 11. (**D**,**E**) Body weight changes over time. (**F**) Final body weight change on day 11. (**G**,**H**) Daily diarrhoeal scores, and (**I**) final diarrhoeal scores on day 11. (**J**,**K**) Daily faecal blood scores, and (**L**) final faecal blood scores on day 11. (**M**,**N**) Daily grimace scores. (**O**) Final grimace score on day 11. Data are expressed as mean ± SEM; statistical significance was determined using one-way ANOVA followed by Dunnett’s multiple comparisons test. * *p* < 0.05, ** *p* < 0.01, *** *p* < 0.001, **** *p* < 0.0001 vs. vehicle DSS control; # *p* < 0.05, ## *p* < 0.01, ### *p* < 0.001, #### *p* < 0.0001 vs. healthy normal water control.

To assess whether treatment with HU308 and CBD could influence the development of severe colitis in a DSS-induced colitis model, mice were continuously administered 4% DSS for 11 days to induce ulcerative colitis. From day one, different doses of HU308 and CBD were given daily for the entire 11-day period. The DAI scores in the vehicle-treated DSS water group increased significantly from day 4 compared to the normal water group. Different doses of CBD and HU308 were used to find the effective dose required to reduce the DAI scores. Treatment with CBD at 60 mg/kg, but not 10 and 30 mg/kg, significantly reduced DAI scores, whereas HU308, at both low and high doses, significantly reduced the DAI scores compared to the vehicle group, which was especially noticeable from day 5 onwards (Figure 2A,B). These significant effects were present till the end of the study as calculated by a change in DAI scores between the final day (day 11) and the baseline (day 1) as shown in Figure 2C (*p* < 0.0001, control vs. vehicle; *p* < 0.01, vehicle vs. 60 mg/kg CBD; *p* < 0.05, vehicle vs. 1 and 10 mg/kg HU308; and *p* < 0.01, vehicle vs. 2.5 mg/kg HU308).

##### HU308 Maintained Healthy Body Weight

The vehicle-treated DSS group mice exhibited significant weight loss compared to the normal water group. CBD treatments had no beneficial effects on weight loss over the study period but HU308, at 1 and 2.5 mg/kg doses, was able to maintain normal body weight as demonstrated by no significant change in weight loss compared to the control water group (*p* > 0.05, Figure 2F).

##### CBD and HU308 Reduced Diarrhoeal Scores

The diarrhoeal scores exhibited a pattern similar to the DAI scores. The DSS water group treated with the vehicle showed significantly higher diarrhoeal scores. However, treatment with CBD and HU308 led to notable improvements. High-dose CBD (60 mg/kg) demonstrated effectiveness throughout the study period, with significant reductions observed from day 7 until the study’s conclusion compared to the vehicle group (*p* < 0.05).

Regarding HU308, the high dose (10 mg/kg) initially showed improvement but failed to maintain a significant reduction until the endpoint. Interestingly, lower doses of 1 and 2.5 mg/kg successfully reduced diarrhoeal scores significantly until the study’s conclusion (*p* < 0.05; Figure 2I).

##### CBD and HU308 Reduced Faecal Blood Scores

Faecal blood scores significantly increased from day 6 and peaked at day 8 in the vehicle-treated colitis group, indicating severe colitis (*p* < 0.05). CBD treatments, mainly 60 mg/kg dose, and HU308 tested at all doses, reduced peaked faecal blood scores to the level of healthy controls. The endpoint faecal blood score changes on day 11, normalized to the healthy control levels, highlighting the protective effects of the treatments.

##### CBD and HU308 Reduced Grimace Scores

The vehicle-treated DSS water group showed significantly higher grimace scores compared to the normal water group starting from day 8 (*p* < 0.001). The CBD treatments had no beneficial effects on the grimace score, whereas HU308, at 2.5 mg/kg dose, significantly reduced the grimace score compared to the vehicle (*p* < 0.001, Figure 2M,N). Figure 2O summarizes these findings, showing the highest efficacy for 2.5 mg/kg HU308.

#### 3.1.2. Impact of CBD and HU308 Treatments on Colon Length, MPO Activity, Plasma GLP-1 Levels, and Spleen Metrics

##### CBD and HU308 Increased Colon Length

**Figure 3:**
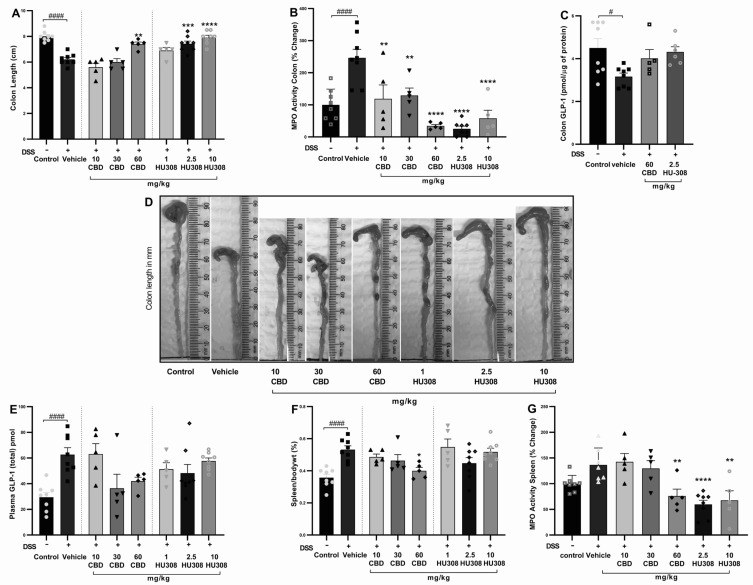
**Effect of CBD and HU308 on colon length, MPO activity, GLP-1 levels, and spleen metrics in DSS-induced colitis**. (**A**) Colon length. (**B**) MPO activity in the colon. (**C**) GLP-1 levels in colon lysates. (**D**) Representative images of colons. (**E**) Plasma GLP-1 levels. (**F**) Spleen to body weight percentage. (**G**) MPO activity in colitis spleen. Data are expressed as mean ± SEM; statistical significance was determined using one-way ANOVA followed by Dunnett’s multiple comparisons test or *t*-test as appropriate. * *p* < 0.05, ** *p* < 0.01, *** *p* < 0.001, **** *p* < 0.0001 vs. vehicle DSS control; # *p* < 0.05, #### *p* < 0.0001 vs. healthy normal water control.

The shortening of colon length resulting from colon injury is often considered a hallmark of DSS-induced colitis. The vehicle-treated DSS group had a significant reduction in colon length compared to the healthy control group (*p* < 0.0001). Treatments with CBD and HU308 ameliorated this DSS-induced colon shortening in a dose-dependent manner. Notably, the highest doses of CBD (60 mg/kg) and HU308 (2.5 and 10 mg/kg) resulted in colon lengths comparable to the healthy control group, demonstrating a significant protective effect against DSS-induced colonic damage (Figure 3A; *p* < 0.01, *p* < 0.001, *p* < 0.0001 vs. vehicle). Representative images of colons from each treatment group are shown in Figure 3D, visually confirming these findings.

##### CBD and HU308 Reduced Colonic MPO Activity

Myeloperoxidase (MPO) activity is a key marker of neutrophil infiltration and acute tissue inflammation. In our study, colonic MPO activity was significantly increased in vehicle-treated colitis compared to the control group (*p* < 0.001), which was associated with colon inflammation. The CBD and HU308 treatments significantly reduced MPO activity, indicating that both compounds exhibit anti-inflammatory properties, with HU308 demonstrating particularly strong effects at lower doses (*p* < 0.0001; Figure 3B).

##### CBD and HU308 Regulated Colon and Plasma GLP-1 Levels

The colonic expression of GLP-1 was significantly reduced in the vehicle-treated colitis group compared to the healthy control animals (*p* < 0.05). While the cannabidiol (CBD) and HU308 treatments showed a trend towards the normalization of GLP-1 levels in colonic tissue, these changes did not reach statistical significance (Figure 3C). In contrast, plasma GLP-1 levels were significantly elevated in the vehicle-treated DSS group compared to the healthy control group (*p* < 0.0001). The treatment with CBD (60 mg/kg) and HU308 exhibited a trend towards the normalization of plasma GLP-1 levels, although these reductions were not statistically significant (Figure 3E). These findings suggest a potential modulatory effect of CBD and HU308 on GLP-1 expression and secretion under inflammatory conditions.

##### CBD and HU308 Demonstrated a Beneficial Role in Spleen Metrics

The elevated spleen weight to body weight ratio, an indicator of splenomegaly and systemic inflammation, is commonly reported in DSS-induced colitis. The DSS treatment resulted in a significant increase in the spleen/body weight ratio percentage compared to the control group (Figure 3F; *p* < 0.0001). Among the treatment groups, only the 60 mg/kg CBD treatment significantly reduced the spleen/body weight ratio (*p* < 0.05 vs. vehicle), indicating a reduction in systemic inflammation and splenomegaly.

##### CBD and HU308 Reduced MPO Activity in the Spleen

The activity of myeloperoxidase in the spleen is measured to further evaluate the extent of systemic inflammation. The elevated MPO activity in the vehicle-treated DSS group is significantly reduced by the CBD treatment at 60 mg/kg dose (*p* < 0.01; Figure 3G). However, HU308 significantly reduced MPO activity at all doses tested. These findings indicate the general anti-inflammatory effect of both CBD and HU308.

#### 3.1.3. Inflammatory Cytokine Levels in DSS-Induced Colitis with CBD and HU308 Treatment

The levels of inflammatory cytokines that are frequently reported to be elevated in colon tissue during colitis were measured to assess the anti-inflammatory effects of CBD and HU308 in DSS-induced colitis.

##### CBD and HU308 Reduced Interleukin-6 (IL-6)

**Figure 4:**
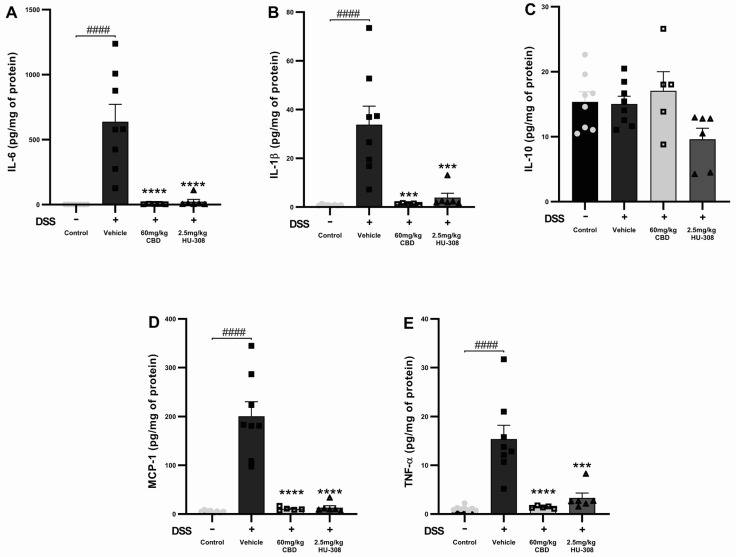
**Effects of CBD and HU308 on inflammatory cytokine levels in DSS-induced colitis.** (**A**) IL-6 levels. (**B**) IL-1β levels. (**C**) IL-10 levels. (**D**) MCP-1 levels. (**E**) TNF-α levels. Data are presented as mean ± SEM; statistical significance was determined using one-way ANOVA followed by Dunnett’s multiple comparisons test. *** *p* < 0.001, **** *p* < 0.0001 vs. vehicle DSS control; #### *p* < 0.0001 vs. healthy normal water control.

The DSS vehicle group had a significant increase in IL-6 levels compared to the healthy control (*p* < 0.0001). Both the 60 mg/kg CBD and 2.5 mg/kg HU308 treatments markedly reduced IL-6 levels to near-baseline levels (*p* < 0.0001 vs. vehicle; Figure 4A).

##### CBD and HU308 Reduced Interleukin-1 Beta (IL-1β)

IL-1β levels were significantly increased in the DSS vehicle group compared to the healthy control groups (*p* < 0.0001). The treatment with 60 mg/kg CBD or 2.5 mg/kg HU308 significantly decreased IL-1β levels, demonstrating strong anti-inflammatory effects (*p* < 0.001 vs. vehicle; Figure 4B).

##### CBD and HU308 Had No Effect on Interleukin-10 (IL-10)

DSS-induced colitis did not significantly alter IL-10 levels compared to the healthy control. The treatments with CBD and HU308 did not produce significant changes in IL-10 levels, indicating that their anti-inflammatory effects do not involve the modulation of IL-10 (Figure 4C).

##### CBD and HU308 Reduced Monocyte Chemoattractant Protein-1 (MCP-1)

DSS administration resulted in a substantial increase in MCP-1 levels in the colitis vehicle group (*p* < 0.0001). Both the 60 mg/kg CBD and 2.5 mg/kg HU308 treatments significantly reduced MCP-1 levels to near-normal levels (*p* < 0.0001 vs. vehicle; Figure 4D).

##### CBD and HU308 Reduced Tumour Necrosis Factor-Alpha (TNF-α)

TNF-α levels were significantly elevated in the colitis vehicle group compared to the healthy controls (*p* < 0.0001). The treatment with 60 mg/kg CBD and 2.5 mg/kg HU308 significantly lowered TNF-α levels compared to the vehicle, indicating potent anti-inflammatory properties (*p* < 0.0001 for CBD; *p* < 0.001 for HU308; Figure 4E).

These results indicate that both CBD and HU308 significantly reduce the levels of pro-inflammatory cytokines IL-6, IL-1β, MCP-1, and TNF-α in DSS-induced colitis, thereby demonstrating their potential as effective anti-inflammatory agents in this model.

#### 3.1.4. Safety Profile of CBD and HU308

**Figure 5:**
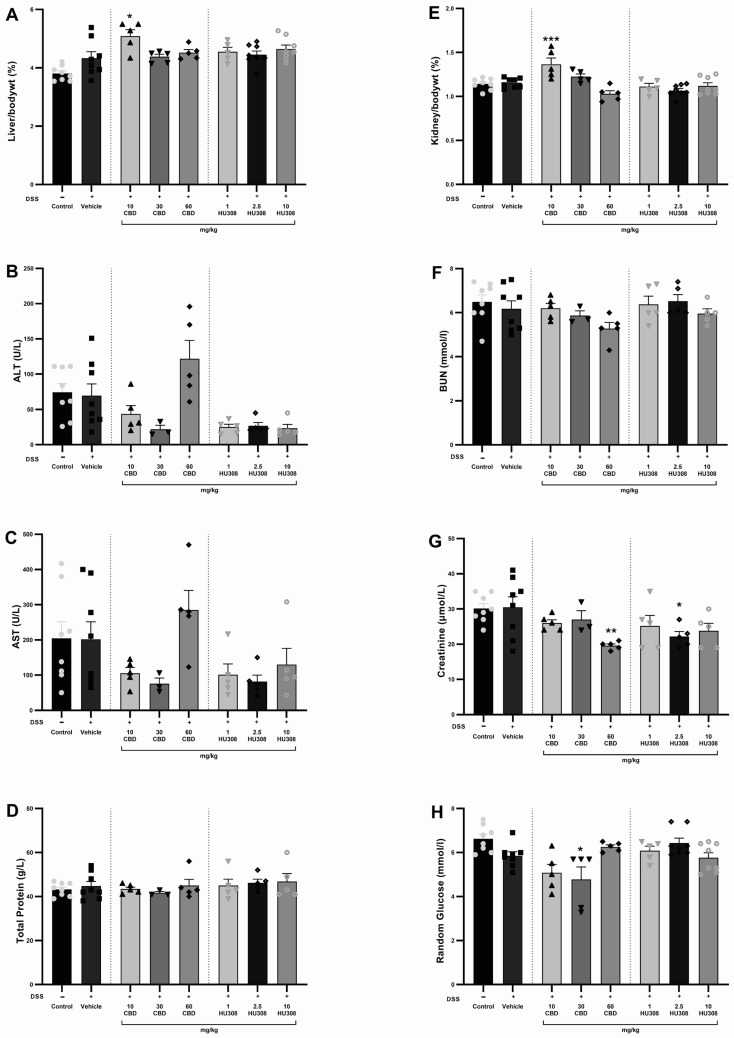
**Assessment of liver and kidney function in DSS-induced colitis**. (**A**) Liver weight to body weight percentage. (**B**) Alanine aminotransferase (ALT) levels. (**C**) Aspartate aminotransferase (AST) levels. (**D**) Total protein levels. (**E**) Kidney to body weight percentage. (**F**) Blood urea nitrogen (BUN) levels. (**G**) Creatinine levels. (**H**) Random glucose levels. Data are presented as mean ± SEM; Statistical significance was determined using one-way ANOVA followed by Dunnett’s multiple comparisons test. * *p* < 0.05 ** *p* < 0.01 *** *p* < 0.001 vs. vehicle DSS control.

The safety of CBD and HU308 in DSS-induced colitis was evaluated by assessing haematology, liver, and kidney function through the analysis of various biochemical parameters and the organ to body weight percentage. There were no significant alterations in haematological parameters in either the colitis or treatment groups compared to the healthy control animals (Table 4). The liver/body weight percentage was significantly increased in the 10 mg/kg CBD group compared to the vehicle-treated colitis group (*p* < 0.05; Figure 5A). There was no significant change in the levels of ALT, AST, and total protein (Figure 5B–D). Similarly, the kidney/body weight percentage increased significantly in the 10 mg/kg CBD treatment group compared to the vehicle-treated colitis group (*p* < 0.001; Figure 5E), whereas the BUN levels were unaffected across the groups (Figure 5F). Creatinine levels were significantly reduced (Figure 5G) with 30 mg/kg CBD (*p* < 0.01) and 10 mg/kg HU308 (*p* < 0.05) compared to the vehicle-treated group. Random glucose levels were significantly reduced with 30 mg/kg CBD (*p* < 0.05).

### 3.2. Results of Chronic Studies

#### 3.2.1. Impact of HU308 Treatment on DSS-Induced Colitis Clinical Markers

##### HU308 Group Maintained Healthy Body Weight Throughout the Study

**Figure 6:**
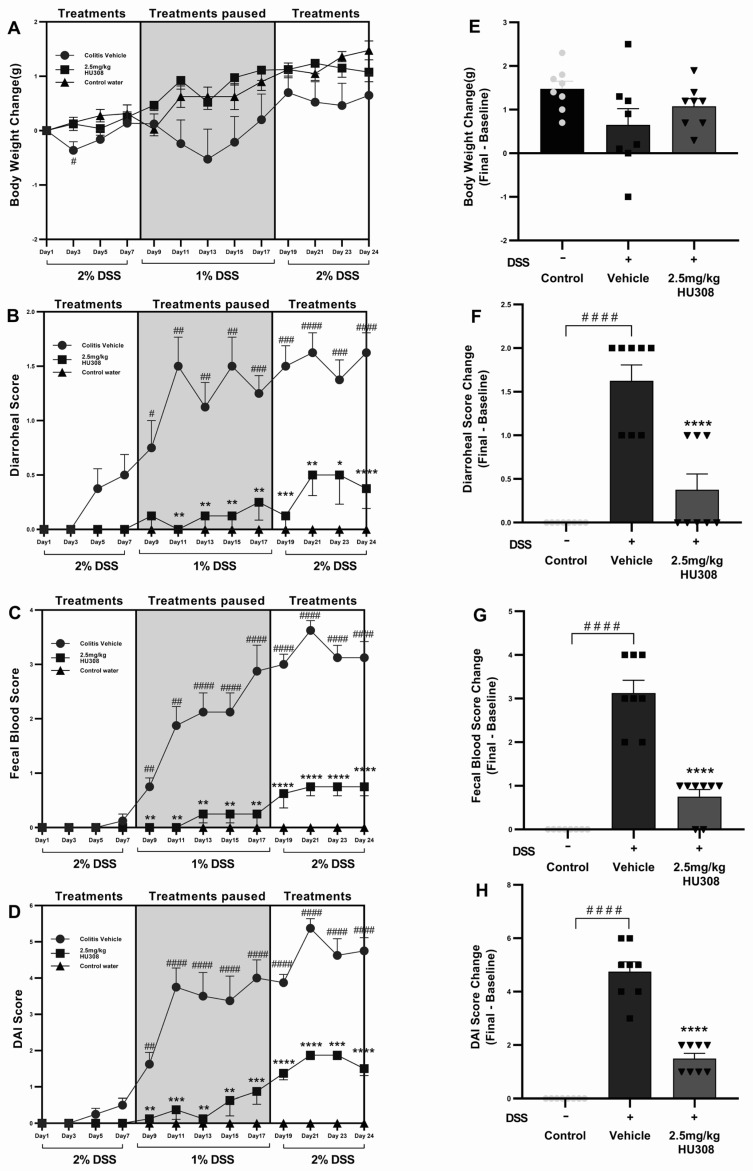
**Effect of HU308 on clinical parameters in DSS-induced chronic colitis.** HU308 treatment significantly mitigated DSS-induced body weight loss (**A**,**E**), diarrhoeal scores (**B**,**F**), faecal blood scores (**C**,**G**), and DAI scores (**D**,**H**) compared to the colitis vehicle group. Data are presented as mean ± SEM, with significant differences indicated by * *p* < 0.05, ** *p* < 0.01, *** *p* < 0.001, **** *p* < 0.0001 compared to vehicle groups and #### *p* < 0.0001, ### *p* < 0.001, ## *p* < 0.01, # *p* < 0.05 compared to control groups. Statistical significance was determined using one-way ANOVA followed by Dunnett’s multiple comparisons test.

DSS administration caused weight loss in the colitis vehicle group compared to the control water group throughout the study period, becoming significant on day 3 (*p* < 0.05; Figure 6A), while the HU308 treatment mitigated this weight loss and brought back the body weight to the level of the healthy control by the end of the study (Figure 6A,E).

##### HU308 Reduced Diarrhoeal Scores

Diarrhoeal scores were significantly elevated in the colitis vehicle group starting from day 7 compared to the healthy control group, indicating severe diarrhoea. The HU308 treatment significantly reduced the diarrhoeal scores from day 8 and maintained the same effect till the end of the study (Figure 6B). HU308 significantly reduced the final change in diarrhoeal scores compared to the vehicle group (*p* < 0.0001; Figure 6F).

##### HU308 Reduced Faecal Blood Scores

The faecal blood score increased significantly in the colitis vehicle group, indicative of colon damage and intestinal bleeding starting from day 9 till the end of the study, while the HU308 treatment markedly reduced these scores (*p* < 0.01, *p* < 0.0001; Figure 6C). The final change in faecal blood score is significantly higher in the vehicle-treated DSS group compared to the healthy control group (*p* < 0.0001) and is lower in the HU308-treated group compared to the vehicle group (*p* < 0.0001; Figure 6G).

##### HU308 Reduced DAI Scores

The DAI scores were significantly higher in the colitis vehicle group starting from day 9, reflecting an increased disease severity (*p* < 0.01–*p* < 0.0001). The HU308 treatment significantly decreased the DAI scores (*p* < 0.05, *p* < 0.001, *p* < 0.0001; Figure 6D). The final change in DAI scores is significantly increased in the DSS vehicle group, whereas the HU308 treatment group had a significant reduction compared to the vehicle group (*p* < 0.0001; Figure 6H).

In summary, the HU308 treatment significantly ameliorated DSS-induced colitis symptoms, as evidenced by reduced levels of body weight loss, diarrhoeal scores, faecal blood scores, and DAI scores. These findings suggest that HU308 has a protective effect against DSS-induced colitis.

#### 3.2.2. Impact of HU308 Treatments on Colon Length, MPO Activity, Plasma GLP-1 Levels, and Spleen Metrics in DSS-Induced Chronic Colitis

To investigate the therapeutic potential of HU308 in DSS-induced chronic colitis, several inflammatory parameters were evaluated, including colon length, spleen/body weight ratio, colon and spleen MPO activity, and GLP-1 levels.

##### HU308 Increased Colon Length

**Figure 7:**
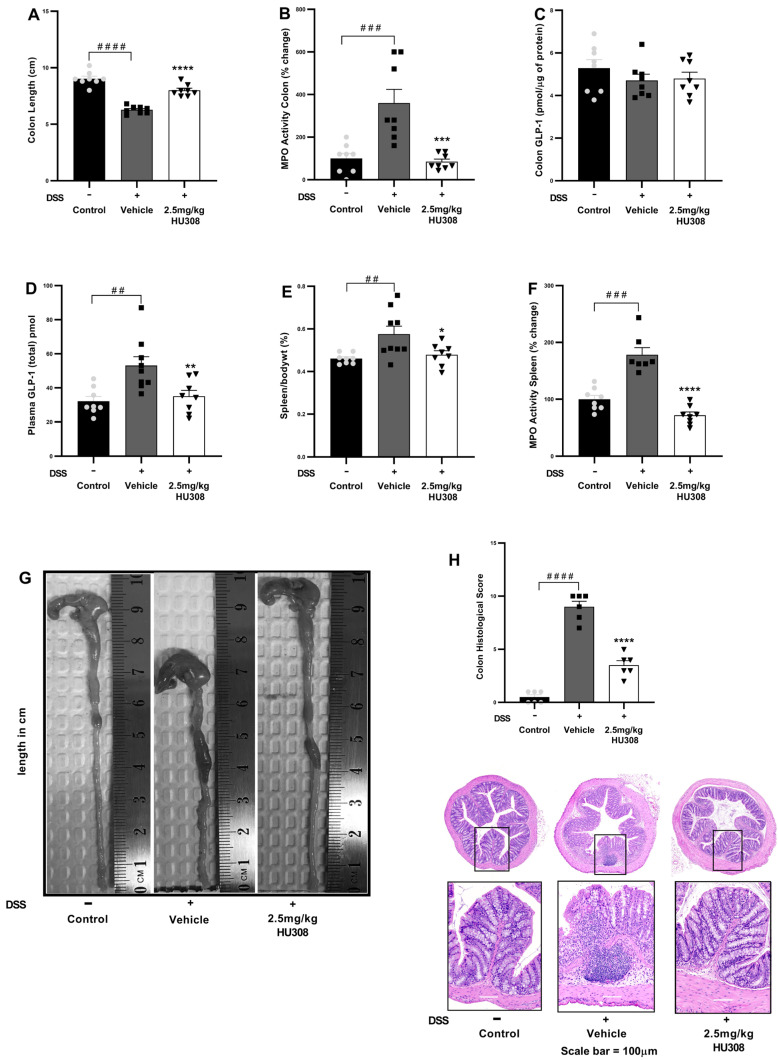
**HU308 ameliorates DSS-induced colitis in mice**. (**A**) Colon length. (**B**) MPO activity in the colon. (**C**) GLP-1 levels in colon lysates. (**D**) Plasma GLP-1 levels. (**E**) Spleen/body weight ratio. (**F**) MPO activity in the spleen. (**G**) Representative images of colons from each treatment group. (**H**) Colon histology score and representation of H and E images. Data are presented as mean ± SEM. #### *p* < 0.0001, ### *p* < 0.001, ## *p* < 0.01, vs. control; * *p* < 0.05, ** *p* < 0.01, *** *p* < 0.001, **** *p* < 0.0001 vs. vehicle.

The vehicle-treated DSS mice, similarly to what was observed in the acute study, exhibited a significant reduction in colon length compared to the healthy controls (*p* < 0.0001). The treatment with 2.5 mg/kg HU308 significantly increased colon length to that found in the healthy controls (*p* < 0.0001; Figure 7A).

##### HU308 Reduced MPO Activity in the Colon

MPO activity, an indicator of neutrophil infiltration, was significantly increased in the colons of the DSS-treated mice compared to the controls (*p* < 0.001). The 2.5 mg/kg HU308 treatment significantly reduced MPO activity compared to the vehicle (*p* < 0.001; Figure 7B).

##### HU308 Regulated Colonic and Plasma GLP-1 Levels

There was no significant change in colonic GLP-1 expressions in both the colitis and treatment groups compared to the healthy controls (Figure 7C). However, the expression of GLP-1 was significantly elevated in the plasma of the vehicle-treated colitis group compared to the healthy controls (*p* < 0.01; Figure 7D). The HU308 treatment significantly reduced plasma GLP-1 levels to normal levels in the healthy mice (*p* < 0.01).

##### HU308 Reduced Relative Spleen Weight

The relative weight of the spleen expressed as the spleen/body weight percentage was significantly increased in the vehicle-treated DSS group compared to the healthy controls (*p* < 0.01). The HU308 treatment significantly reduced it compared to the vehicle-treated DSS mice (*p* < 0.05; Figure 7E).

##### HU308 Reduced MPO Activity in the Spleen

MPO activity in the spleen was significantly increased in the vehicle-treated DSS group compared to the healthy control (*p* < 0.001). The HU308 treatment significantly reduced MPO activity in the spleen compared to the vehicle (*p* < 0.0001; Figure 7F).

##### HU308 Normalized Colon Histopathological Score

The shortening of the colon in the vehicle-treated DSS group was ameliorated by the HU308 treatment as demonstrated by representative colon images (Figure 7G). Similarly, increased histopathological scores were evident in the vehicle-treated DSS group, which were reduced by the HU308 treatment (*p* < 0.0001; Figure 7H). The data demonstrate that HU308 has a significant therapeutic effect on DSS-induced colitis in mice, as evidenced by the restoration of colon length, reduction in MPO activity in both the colon and spleen, normalization of plasma GLP-1 levels, and reduction in spleen/body weight ratio. These findings suggest that HU308 may be a potential therapeutic agent for inflammatory bowel disease (IBD).

#### 3.2.3. HU308 Reduced Pro-Inflammatory Cytokine Levels in DSS-Induced Colitis

**Figure 8:**
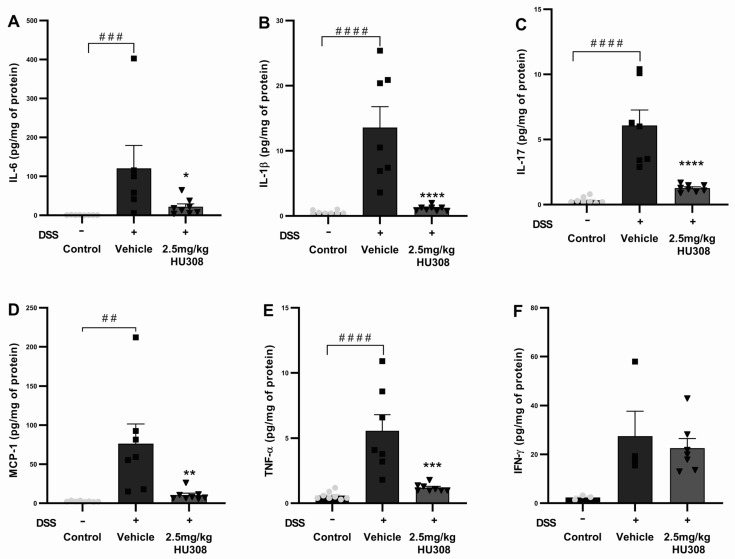
**HU308 reduces pro-inflammatory cytokine levels in DSS-induced colitis.** (**A**) IL-6 levels. (**B**) IL-1β levels. (**C**) IL-17 levels. (**D**) MCP-1 levels. (**E**) TNF-α levels. (**F**) IFN-γ levels. Data are presented as mean ± SEM. * *p* < 0.05, ** *p* < 0.01, *** *p* < 0.001, **** *p* < 0.0001 vs. vehicle; ## *p* < 0.01, ### *p* < 0.001, #### *p* < 0.0001 vs. control. Statistical significance was determined using one-way ANOVA followed by Dunnett’s multiple comparisons test.

To further elucidate the anti-inflammatory effects of HU308 in DSS-induced colitis, we measured the levels of several pro-inflammatory cytokines in chronic colon tissues, including IL-6, IL-1β, IL-17, MCP-1, TNF-α, and IFN-γ. The DSS treatment significantly increased IL-6 levels in the colon tissues of the vehicle group compared to those of the healthy control (*p* < 0.001), which were significantly reduced by the HU308 treatment (*p* < 0.05; Figure 8A). The IL-1β levels were significantly elevated in the vehicle-treated DSS group compared to those of the healthy control (*p* < 0.0001), which were significantly reduced by the HU308 treatment to the level of the healthy control (*p* < 0.0001; Figure 8B). The DSS treatment caused a significant increase in IL-17 levels in the vehicle-treated group compared to the healthy control (*p* < 0.0001). The HU308 treatment markedly reduced the IL-17 level to the level of the healthy control group (*p* < 0.0001; Figure 8C). MCP-1 levels were significantly elevated in the vehicle-treated DSS group compared to those of the healthy control (*p* < 0.01), which were significantly reduced by the HU308 treatment (*p* < 0.01; Figure 8D). Similarly, the DSS treatment significantly increased TNF-α levels in the vehicle-treated group compared to those of the healthy control (*p* < 0.0001), which were significantly reduced by the HU308 treatment to the level of the healthy control (*p* < 0.001; Figure 8E). There was no significant change in the level of IFN-γ among the groups tested (*p* > 0.05; Figure 8F).

The data indicate that HU308 significantly reduces the levels of several pro-inflammatory cytokines, including IL-6, IL-1β, IL-17, MCP-1, and TNF-α, in the colon tissues of DSS-treated mice. These findings suggest that HU308 exerts potent anti-inflammatory effects in the context of DSS-induced chronic colitis.

#### 3.2.4. Impact of HU308 Treatment on Haematology and Liver and Kidney Function

**Figure 9:**
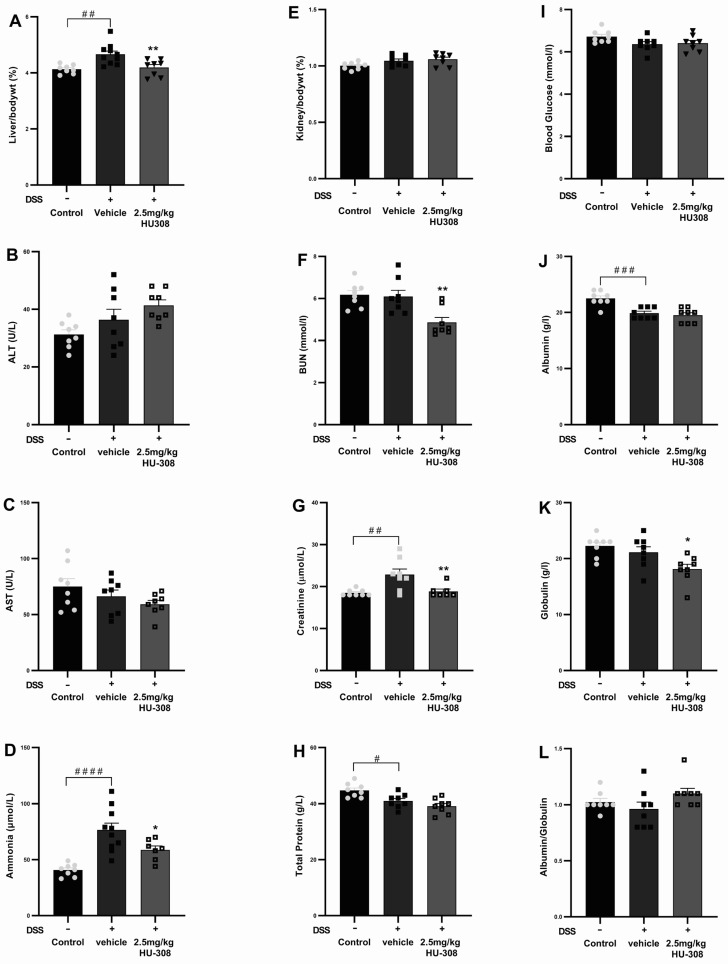
**Effect of HU308 on liver and kidney function in DSS-induced colitis.** (**A**) Liver to body weight ratio. (**B**) Alanine aminotransferase (ALT) levels. (**C**) Aspartate aminotransferase (AST) levels. (**D**) Ammonia levels. (**E**) Kidney to body weight ratio. (**F**) Blood urea nitrogen (BUN) levels. (**G**) Creatinine levels. (**H**) Total protein levels. (**I**) Random blood glucose levels. (**J**) Albumin levels. (**K**) Globulin levels. (**L**) Albumin/globulin ratio. Data are presented as mean ± SEM. Significant differences are indicated by * *p* < 0.05, ** *p* < 0.01 compared to vehicle, and # *p* < 0.05 ## *p* < 0.01, ### *p* < 0.001, #### *p* < 0.0001 compared to control. Statistical significance was determined using one-way ANOVA followed by Dunnett’s multiple comparisons test.

There was no significant change in haematological parameters in both the colitis group and the HU308 treatment group except for the WBC and lymphocytes, which were significantly reduced in the HU308 treatment group compared to the healthy control group (*p* < 0.05; Table 5). However, the administration of HU308 significantly reduced various biochemical and physiological parameters in the DSS-induced colitis model, as illustrated in Figure 9. The liver to body weight percentage was significantly increased in the DSS-induced vehicle-treated colitis group compared to the normal controls (*p* < 0.01). This was significantly reduced by the HU308 treatment at 2.5 mg/kg, restoring it to the level observed in the healthy control group (*p* < 0.01; Figure 9A). Plasma levels of ALT and AST (Figure 9B,C) were not affected in both DSS-induced colitis and HU308 treatment groups. The plasma ammonia and creatinine levels were significantly increased in the vehicle-treated DSS group compared to the healthy normal group (*p* < 0.0001 and *p* < 0.01, respectively); these values were brought back to the healthy control levels by the HU308 treatment (*p* < 0.05 and *p* < 0.01, respectively; Figure 9D,G), indicating its protective effects on liver and renal function, respectively. Furthermore, the HU308 treatment significantly decreased BUN levels (Figure 9F) and maintained stable total protein levels (Figure 9H), further suggesting its renoprotective benefits. While the kidney to body weight percentage (Figure 9E), blood glucose levels (Figure 9I), albumin levels (Figure 9J), and albumin/globulin ratio (Figure 9L) did not show significant changes, the globulin level was significantly reduced by the HU308 treatment (*p* < 0.05; Figure 9K). The overall findings of the biochemical and physiological parameters underscore the beneficial effects of HU308 on both liver and kidney function in the context of DSS-induced colitis.

## 4. Discussion

Inflammatory bowel diseases, including ulcerative colitis, are characterized by clinical symptoms such as diarrhoea, faecal blood, weight loss, and abdominal pain, which substantially diminish patients’ quality of life [32]. Current therapeutic options for ulcerative colitis are limited by their intolerable side effects, diminishing efficacy over time, and failure to address the extraintestinal manifestations of the disease. To address these gaps, we employed a novel comprehensive approach to evaluate both the local and systemic effects of CBD and HU308 in DSS-induced colitis. We used the DSS-induced colitis model as it has been widely utilized by researchers due to its simplicity, reproducibility, and strong resemblance to human ulcerative colitis, making it a valuable tool for the preclinical evaluation of novel therapeutics. Its capacity to closely mimic the pathophysiology of human disease enhances the translational relevance of experimental findings to clinical contexts. By adjusting DSS concentration and administration protocols, it is easy to precisely modulate the severity and duration of inflammation, enabling detailed investigations into both acute and chronic colitis [22]. Furthermore, the rapid onset of symptoms in this model supports the efficient screening of therapeutic candidates. Overall, the DSS model serves as a robust and well-characterized platform for exploring immunomodulatory therapies pertinent to inflammatory bowel diseases. We used both acute and chronic mouse models of DSS-induced colitis to explore the therapeutic potential of CBD and HU308.

In the acute DSS-induced colitis model, CBD and HU308 demonstrated significant efficacy in ameliorating clinical symptoms and underlying pathophysiology. Treatment with CBD (60 mg/kg) and HU308 (2.5 mg/kg) resulted in marked reductions in disease activity index (DAI) scores, diarrhoeal scores, body weight loss, faecal blood, and grimace scores. Notably, HU308 normalized all clinical markers to levels comparable to those of healthy controls at a substantially lower dose than CBD (Figure 2).

These clinical improvements were corroborated by beneficial effects on colitis pathophysiology. Both CBD and HU308 restored colon length and reduced colonic myeloperoxidase (MPO) activity (Figure 3), established markers of intestinal damage and inflammation [22,25]. Furthermore, the treatments normalized spleen to body weight ratios, splenic MPO activity, and GLP-1 levels, indicating the mitigation of extraintestinal manifestations of colitis. GLP-1 plays a crucial role beyond its anti-diabesity function, as it has been shown to be dysregulated in inflammatory conditions, including those that disrupt gut barrier functions [33]. Our study revealed a complex pattern of GLP-1 expression during colitis: decreased levels in the colon but increased concentrations in plasma. The reduced colonic GLP-1 level likely indicates damage to L cells, which are responsible for GLP-1 production. Conversely, the elevated plasma GLP-1 levels may suggest compensatory secretion from areas outside the colon, such as the small intestine. The observed reduction in colonic GLP-1 expression in the colitis group is consistent with previous studies showing decreased GLP-1 receptor mRNA in inflamed colonic tissues of IBD patients [34]. Conversely, the elevated plasma GLP-1 levels in the DSS-induced colitis group corroborate earlier findings of increased serum GLP-1 in IBD patients [34]. The trend towards the normalization of both colonic and plasma GLP-1 levels with the CBD and HU308 treatments suggests the potential anti-inflammatory effect of these compounds, possibly mediated through GLP-1 signalling pathways [35,36].

Remarkably, the treatments with CBD (cannabidiol) and HU308 appeared to normalize plasma GLP-1 levels, bringing them closer to those observed in healthy controls. These elevated plasma GLP-1 levels in colitis corresponded with a reduction in blood glucose levels compared to healthy controls. Following treatment, both plasma GLP-1 and blood glucose levels were normalized to those of healthy controls. This finding underscores the potential therapeutic effects of these compounds on GLP-1 regulation during inflammatory conditions. Our findings corroborate existing research identifying GLP-1 as a versatile anti-inflammatory agent across various disorders [31,35,37], which supports its importance in maintaining gut health and regulating inflammatory responses. GLP-1 modulates inflammatory cytokine expression at local and systemic levels [38,39]. We observed significant increases in colonic IL-6, IL-1β, MCP-1, and TNF-α levels in colitis, which were effectively reduced by CBD and HU308 treatments (Figure 4). These cytokines play critical roles in the pathogenesis of colitis, with IL-6, TNF-α, and IL-1β promoting immune cell recruitment and tissue damage, while MCP-1 contributes to macrophage activation and mucosal injury [40,41].

Safety profiling through haematological (Table 4), hepatic, and renal function analyses revealed no significant alterations in liver to body weight ratio (%), kidney to body weight ratio (%), ALT, AST, BUN, and total protein levels across treatment groups (Figure 5). However, a significant reduction in serum creatinine levels was observed in the groups receiving 60 mg/kg CBD and 2.5 mg/kg HU308 compared to the colitis group. These findings suggest that the treatments do not induce notable hepatotoxicity or nephrotoxicity.

Consistent with the superior efficacy of HU308 (2.5 mg/kg) over CBD (60 mg/kg) in acute colitis, we extended our investigation to assess the long-term efficacy of HU308 using a chronic model of colitis. The HU308 treatment significantly ameliorated clinical markers of chronic colitis, evidenced by reduced levels of body weight loss, diarrhoeal scores, faecal blood scores, and DAI scores (Figure 6). These findings suggest that HU308 maintains prolonged beneficial effects in chronic colitis. The reduction in clinical markers was supported by improvements in the underlying pathophysiology of colitis. The HU308 treatment restored colon length and reduced colonic MPO activity, indicating reduced intestinal inflammation and damage. Like acute colitis, chronic colitis was associated with significant increases in plasma GLP-1 but no significant change in colonic levels, spleen-to body weight percentage, or spleen MPO activity, indicating extraintestinal involvement. The HU308 treatment normalized these extraintestinal markers to levels comparable with healthy controls, further supporting the clinical markers (Figure 7).

Similar to the acute model, HU308 significantly reduced the colonic expression of pro-inflammatory cytokines and chemokines, including IL-6, IL-1β, MCP-1, and TNF-α, to levels comparable to those in healthy controls (Figure 8). The reduction in these markers by HU308 aligns with previous studies showing the therapeutic potential of cannabinoids in modulating inflammatory responses in IBD [25,42,43]. In the acute colitis model, we initially aimed to assess the impact of DSS on anti-inflammatory cytokines as well, particularly IL-10, given its pivotal role in immune regulation. However, our results did not show significant changes in IL-10 expression between healthy controls and vehicle-treated colitis groups or in response to our treatments. Consequently, we shifted our focus to IL-17 for the chronic colitis model. IL-17 plays a critical role in the development and perpetuation of IBD due to its potent pro-inflammatory effects [44]. It has been consistently shown to be significantly elevated in patients with active colitis [45]. In line with these findings, our chronic colitis model demonstrated a marked elevation of IL-17 levels compared to those of healthy animals. Notably, this elevation was significantly attenuated by our treatments, highlighting their potential therapeutic impact. This suggests that HU308 may mitigate inflammation and aid in tissue repair, highlighting its potential as a novel therapeutic for colitis. HU308 has been investigated in several models of inflammatory disorders, in which it has demonstrated strong anti-inflammatory effects, including corneal pain and inflammation [7], proliferative vitreoretinopathy [19], uveitis [46,47], sepsis [48], diabetic cardiomyopathy [49], hepatic ischemia [50,51], etc., all via the modulation of CB2 receptors. Additionally, HU308, via the modulation of CB2 receptors, was shown to reduce DSS-induced colitis by inhibiting the nucleotide-binding domain and leucine-rich repeat protein 3 (NLRP3) inflammasome in macrophages [42]. Here, we have explored other possible mechanisms including its role in GLP-1 and ammonia modulation.

Our investigation into the long-term safety profile of HU308 revealed significant improvements in both haematological parameters (Table 5) and hepatic and renal functions (Figure 9). Chronic colitis induced a significant increase in relative liver weight, which was attenuated by HU308 treatment, suggesting hepatoprotective properties. Furthermore, the marked reduction in blood BUN and creatinine levels indicates a beneficial effect on renal function. Notably, the significant elevation in plasma ammonia levels observed in colitis may reflect increased ammonia production in the gut or the disruption of the gut–liver axis. Further investigation in experimental colitis models could offer deeper insights into the role of the ammonia/urea cycle in the liver, where ammonia is normally converted to urea and subsequently excreted in the urine [52]. Elevated ammonia levels have been reported in various central nervous system disorders, including depression, anxiety, and Alzheimer’s disease, which are often associated with chronic colitis as extraintestinal manifestations [53,54,55,56]. Our preclinical data, with the ability of HU308 to significantly reduce ammonia levels, present a potential novel therapeutic approach for addressing these extraintestinal manifestations of colitis. These findings warrant further clinical investigation. Additionally, ammonia levels could be explored as a potential biomarker in clinical settings for colitis.

## 5. Conclusions

In conclusion, the findings of this study underscore the remarkable therapeutic potential of HU308 as compared to cannabidiol in mitigating ulcerative colitis. Through a meticulous evaluation in a mouse model of DSS-induced colitis, we have demonstrated that HU308 exhibits a superior efficacy at lower doses, surpassing the effects achieved by higher doses of CBD. The ability of HU308 to mitigate colitis symptoms and reduce inflammation at local and systemic levels without notable adverse effects holds profound implications for clinical translation, offering a promising avenue for enhanced efficacy and minimising risks in the management of ulcerative colitis. These findings shed light on the therapeutic superiority of HU308 and underscore its potential as a novel treatment modality for ulcerative colitis. Given the effectiveness and safety profile of HU308, future research could explore its clinical utility in human ulcerative colitis.

## Figures and Tables

**Table 1:** Assessment of DAI score.

Clinical Colitis Scores	0	1	2	3
Body weight loss score	<5%	5–10%	11–15%	16–20%
Diarrhoeal score/stool consistency	Normal	Mild-soft, but still formed	Very soft/Sticky	Loose/diarrhoea
Faecal blood score/rectal bleeding	Normal colour stool/no rectal bleeding	Positive hemoccult—slight (brown colour)/slight blood spotting in the anus	Positive hemoccult—darker (reddish)/significant presence of blood in the anus	Visible trace of blood/rectal bleeding

Assessment of DAI score. DAI score is expressed as the sum of body weight loss score, diarrhoea/stool consistency, and faecal blood score/rectal bleeding.

**Table 2:** Assessment of the Grimace score.

Pain Features/Scores	0	1	2
Orbital tightening	Not present	Closing of eyelid, narrowing of orbital area	Complete closure of eye with tightened orbital
Nose bulge	Not present	Slight bulging on the bridge of nose	Completely bulged nose
Cheek bulge	Not present	Slight bulging of the cheek	Completely bulged
Ear position	Normal position	Ears moving towards the back	Folded ear forming a pointed shape
Whisker change	Normal whisker position	Whiskers pulled back/front	Clumping of whiskers
Movement/gait	Normal activity	Moves slowly	Moves only when provoked
Body position/hunching	Normal position	Slight tuck to abdomen	Fully hunched

Assessment of the Grimace score. Grimace score is calculated as average of all 7 parameters listed in column 1.

**Table 3:** Assessment of colon histological score.

Score	Severity of Inflammation	Crypt Damage	Goblet Cell Loss	Ulceration
0	Absent	intact crypts	absent	absent
1	Increased presence of inflammatory cells in lamina propria	Loss of the basal one-third	<10%	
2	Also infiltrates submucosa	Loss of the basal two-thirds	Decreased by 10–50%	
3	Transmural	Loss of entire crypt	Decreased by > 50%	Present

Assessment of colon histological score. Total score is the sum of all individual parameters.

**Table 4:** Effect of HU308 on blood haematology in DSS-induced acute colitis following 11 days repeated daily treatment through intraperitoneal administration.

Treatment Groups	Healthy Control	Colitis Vehicle	2.5 mg/kg HU308	10 mg/kg HU308
**WBC (10^9^/L)**	6.2 ± 0.6	6.8 ± 2.0	4.0 ± 0.3	6.6 ± 1.1
**Lymphocytes (10^9^/L)**	2.9 ± 1.2	5.2 ± 1.7	1.2 ± 1.1	5.2 ± 1.1
**Monocytes (10^9^/L)**	0.1 ± 0.03	0.3 ± 0.1	0.1 ± 0.05	0.3 ± 0.03
**Granulocytes (10^9^/L)**	3.2 ± 1.0	1.3 ± 0.3	2.6 ± 1.0	1.1 ± 0.1
**RBC (10^12^/L)**	9.2 ± 0.1	9.6 ± 0.2	9.4 ± 0.2	9.4 ± 0.2
**Haemoglobin (g/L)**	134.7 ± 2.3	135.4 ± 1.8	137.3 ± 4.3	135.38 ± 2.6
**Haematocrit (%)**	41.9 ± 0.6	43.4 ± 1.0	42.7 ± 1.0	42.6 ± 0.7
**Mean corpuscular volume (fL)**	45.6 ± 0.2	45.2 ± 0.3	45.3 ± 0.4	45.4 ± 0.1
**Mean corpuscular haemoglobin (pg)**	14.6 ± 0.1	14.5 ± 0.1	14.5 ± 0.2	14.4 ± 0.1
**Mean corpuscular haemoglobin concentration (g/L)**	320.8 ± 2.6	321.4 ± 2.4	321.0 ± 2.9	317.3 ± 1.5
**Red cell distribution width (%)**	11.8 ± 0.2	11.6 ± 0.3	11.8 ± 0.2	12.1 ± 0.1
**Platelets (10^9^/L)**	1017.3 ± 65.1	917.4 ± 118.0	1054.0 ± 49	966.3 ± 17.2
**Mean platelet volume (fL)**	4.9 ± 0.02	5.2 ± 0.1	5.4 ± 0.3	5.2 ± 0.1
**Platelet distribution width**	16.3 ± 0.01	16.4 ± 0.1	16.6 ± 0.6	16.2 ± 0.1
**Procalcitonin (%)**	0.5 ± 0.01	0.5 ± 0.1	0.5 ± 0.02	0.5 ± 0.02

Data are expressed in MEAN ± SEM. No statistical differences among the groups.

**Table 5:** Effect of HU308 on blood haematology in DSS-induced chronic colitis.

Treatment Groups	Healthy Control	Colitis Control	2.5 mg/kg HU308
**WBC (10^9^/L)**	5.6 ± 0.8	6.8 ± 0.8	4.4 ± 0.4 *
**Lymphocytes (10^9^/L)**	2.5 ± 1.2	2.4 ± 1.1	0.1 ± 0.04 *
**Monocytes (10^9^/L)**	0.2 ± 0.04	0.2± 0.1	0.1 ± 0.02
**Granulocytes (10^9^/L)**	3.0 ± 1.0	4.2 ± 1.1	4.2 ± 0.4
**RBC (10^12^/L)**	9.2 ± 0.1	8.9 ± 0.1	8.7 ± 0.2
**Haemoglobin (g/L)**	135.3 ± 2.2	131.3 ± 1.3	128.5 ± 2.7
**Haematocrit (%)**	42.3 ± 0.7	41.0 ± 0.3	39.8 ± 0.8
**Mean corpuscular volume (fL)**	45.8 ± 0.2	46.1 ± 0.3	45.8 ± 0.2
**Mean corpuscular haemoglobin (pg)**	14.6 ± 0.1	14.7 ± 0.1	14.7 ± 0.1
**Mean corpuscular haemoglobin concentration (g/L)**	319.3 ± 2.9	319.5 ± 2.4	322.8 ± 1.9
**Red cell distribution width (%)**	11.7 ± 0.2	12.3 ± 0.3	12.5 ± 0.3
**Platelets (10^9^/L)**	1034.3 ± 62.2	980.0 ± 45.8	958.2 ± 26.6
**Mean platelet volume (fL)**	4.9 ± 0.04	4.9 ± 0.9	5.0 ± 0.07
**Platelet distribution width**	16.3 ± 0.05	16.1 ± 0.05	16.1 ± 0.06

Data are presented as mean ± SEM; statistically significant differences are indicated by * *p* < 0.05 compared to vehicle. Statistical significance was determined using one-way ANOVA followed by Dunnet’s multiple comparisons test.

## Data Availability

The original contributions presented in the study are included in the article. Data will be available upon request and further inquiries can be directed to the corresponding authors.

## Abbreviations

ALT: Alanine Aminotransferase; AST: Aspartate Aminotransferase; BUN: Blood Urea Nitrogen; CBD: Cannabidiol; CB2: Cannabinoid 2; DAI: Disease Activity Index; DMSO: Dimethyl Sulfoxide; DSS: Dextran Sodium Sulphate; GI: Gastrointestinal; GLP-1: Glucagon-like Peptide-1; GPR55: G Protein-Coupled Receptor 55; H and E: Hematoxylin and Eosin; IBD: Inflammatory Bowel Disease; IL: Interleukin; MCP: Monocyte Chemoattractant Protein-1; MPO: Myeloperoxidase; PBS: Phosphate Buffered Saline; RIPA: Radioimmunoprecipitation; TNF-α: Tumour Necrosis Factor-Alpha; UC: Ulcerative Colitis.
